# Self-extracted corn-stalk cellulose/epoxy resin composites

**DOI:** 10.1038/s41598-022-25695-0

**Published:** 2022-12-05

**Authors:** Chunhua Lou, Siyu Jiang, An Yan, Yongli Zhou, Yang Liu, Yong Zhang, Xianzhi Kong

**Affiliations:** 1 School of Chemistry and Chemical Engineering, Qiqihar University, Qiqihar, 161006 China; 2 Heilongjiang Province Key Laboratory of Polymer-Based Composites, Qiqihar University, Qiqihar, 161006 China; 3 Institute of Petrochemistry, Heilongjiang Academy of Sciences, Harbin, 150040 China

**Keywords:** Chemical modification, Renewable energy

## Abstract

In order to make full use of crop waste stalk, corn-stalk cellulose (CSC) was extracted by acid–base method and used as modifier of epoxy resin (E51) to prepare the self-extracted corn-stalk cellulose/epoxy resin composites (CSCEC). Differential scanning calorimeter (DSC), thermogravimetry (TG) analysis, dynamic mechanical analysis (DMA), morphology analysis by scanning electron microscope (SEM), the mechanical properties by electronic universal testing machine and impact testing machine were used for characterization and analysis. The experimental results showed that when the CSC content was 20 wt%, the impact strength of the composite was 2.50 kJ/m^2^, which was 127.2% higher than that of pure epoxy resin. When the CSC content was 20 wt%, the Tg of epoxy resin obtained by DMA was the lowest, 167.4 °C, which decreased by 11.3 °C compared with that of pure epoxy resin. The SEM result showed that the fracture surface of the composite became obviously rough and had of obvious folds, which was a ductile fracture. These results indicated that the addition of CSC could toughen the epoxy resin.

## Introduction

The cured epoxy resin has excellent anticorrosion, insulation, thermal stability and high adhesion, and is widely used in electronic devices, coatings, adhesives and composite materials. However, epoxy resin is of high brittleness resulting from its high curing crosslinking density and high internal stress, which limits its application in some high-tech fields. Toughened epoxy resin is always a hot topic in epoxy resin research^1–4^. The common method of toughening epoxy resin is to add toughening agent, such as nano particles^5–7^, liquid crystal polymer^8^, fiber^9,10^, polymer^11,12^, Lignin^13^, block polymer^14,15^, hyperbranched polymer^16^, and natural fills^17–21^ etc., which can effectively improve the brittleness of epoxy resin, and broaden its application in the industrial field. Biomass toughened epoxy resin has become a research hotspot due to its green and sustainable development^22–24^.

Corn-stalk (CS) is a kind of abundant biomass resource. Heilongjiang province of China is a major province in corn production, which produces a large amount of corn stalks every year. However, local farmers have very low utilization rate of corn stalks, and most of them choose direct incineration, which pollutes the environment and wastes a large amount of biomass resources.

Because there are a lot of hydroxyl groups and active groups on its surface, corn stalk shows strong hydrophilicity and polarity, which is not conducive to compatibility with epoxy resin matrix. After being extracted cellulose from corn stalk, the hydroxyl content on its surface can be greatly reduced, thus reducing hydrophilicity^25^. Compared with corn stalk, corn-stalk cellulose has rougher surface and larger specific surface area, which is conducive to the penetration of epoxy resin, forming “glue nails” to increase the mechanical interlocking position and produce stronger mechanical coupling effect, and improving the mechanical properties of composites.

Finding a way to use the corn-stalk has become the focus of our works. For a more useful literature review about cellulose extraction by acid–base method, please review the paper that have already been published^26^. In this paper, the extracted cellulose was used as filler to modify epoxy resin. The thermal stability, dynamic mechanical properties and mechanical properties of the composites were studied, which provided theoretical basis for further application of the composites. In this way, crop waste can be used effectively, which not only protects the environment but also saves energy. Therefore, the novelty of this paper lies in using crop waste extract as the filler of epoxy resin, which belongs to the environment-friendly filler. And the research would be in line with the idea of sustainable development.

## Experimental

### Materials

The epoxy resin diglycidyl ether of bisphenol A (E-51) was purchased from Baling Branch of Sinopec. Corn-stalk was obtained from Qiqihar, Heilongjiang Province in China. The samples were washed and got rid of silt with distilled water were smashed to powder and the diameter was less than 0.178 micron after drying. The dried CS powder was first pretreated with immobilized enzyme in 0.1 mol/L acetic acid/sodium acetate buffer solution at 40 °C for 12 h. The residue was suction filtrated and dried and then subsequently treated with NaOH solution at 60 °C for 2 h and the mixed solution of sodium chlorite and acetic acid at 70 °C for 1 h. The cellulose content of corn-stalk cellulose was 96.72%. Curing agent 2-methyl imidazole (2-MI) was purchased from Tianjin Kermel chemical reagent development center.

### Preparation of CSC/epoxy resin composites

**Figure 1:**
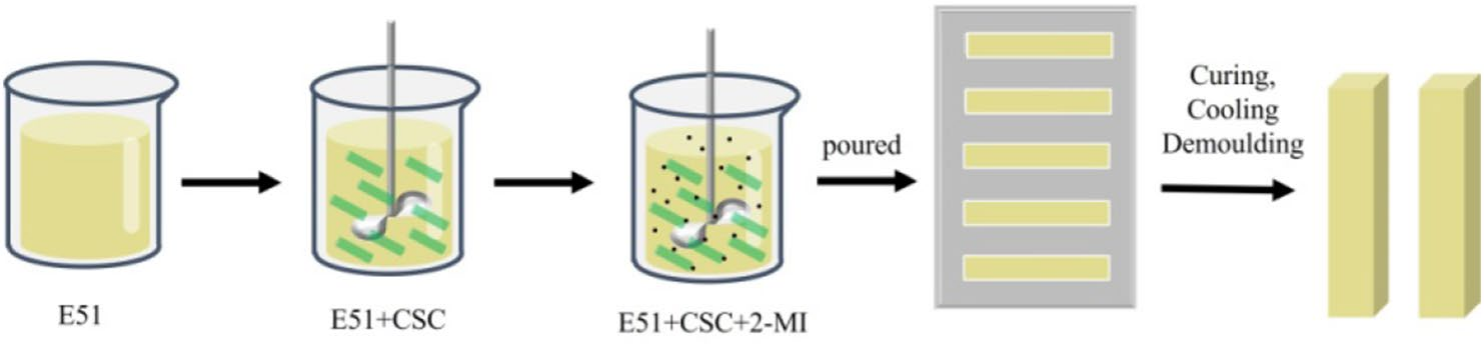
The process flow diagram of preparation of CSCECs.

The extracted CSC was dried in the oven at 80 °C for 24 h. CSC was mixed with epoxy resin in the proportions of 0 wt%, 5 wt%, 10 wt%, 15 wt%, 20 wt%, and 25 wt% in a plastic cup and stirred fully to make CSC dispersed homogeneously in the epoxy resin matrix. Next, 4 wt% 2-MI was added into the six plastic cups, which were stirred evenly. The mixture was then poured into the mold and cured at 80 °C for 2 h. Thereby, the composites were obtained, which were labeled as CSCEC-wt%. For example, when the content of CSC was 5 wt%, the composite was labeled as CSCEC-5. The process flow diagram was shown in Fig. 1.

### Characterization and Testing

FTIR spectra of samples were recorded on a FTIR in a range of wave numbers from 4000 to 500 cm^−1^, using a Frontier Fourier transform infrared (PE Instruments).

Differential scanning calorimetry (DSC) was used to test the exothermic reaction of the composites at the heating rate of 10 K/min by Netzsch DSC 204 F1 differential scanning calorimeter.

Thermogravimetry analyses (TGA) was used to investigate thermal behavior of the composites by TGA5500 thermogravimetric analyzer, Waters Science and Technology Co. Ltd, USA.

Dynamic mechanical analysis (DMA) was used to record the composites’ temperature-dependent visco-elastic properties by Q800 thermomechanical analyzer, TA Instruments.

The impact fracture surface morphology of the composites was undertaken by TM3030 type Scanning Electron Microscope (Hitachi, Japan).

Impact property: According to ISO 179-1:2000, the impact property was conducted on the charpy impact tester (GT-7045-HMH impact testing machine, Gotech Testing Machines Inc., China) at room temperature. The dimension of specimens was 80 × 10 × 4 mm with no notches.

Tensile property: The tensile property was operated on the electronic universal testing machine (WSM-20KN electronic universal testing machine, Changchun Intelligent Instrument Equipment Co., Ltd., China) in accordance with ISO 527:1993 with the loading speed is 2 mm/min at room temperature under the load of 20 kN.

Flexural property: The flexural property was operated on the electronic universal testing machine according to ISO 178:1993 with the loading speed is 2 mm/min at room temperature under the load of 500 N.

## Results and discussion

### FTIR analysis

**Figure 2:**
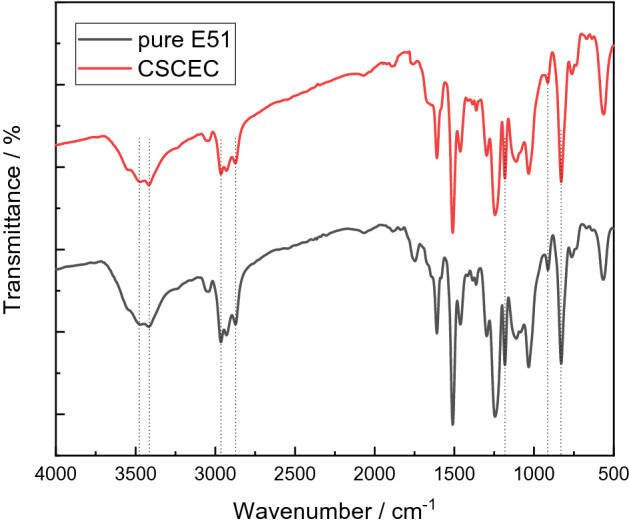
FTIR spectra of curing systems of pure E51 and CSCEC.

Figure 2 showed that the FTIR spectra of the pure epoxy resin and the composites when the addition amount of CSC was 5% (CSCEC-5). The stretching vibration peaks of − OH were at 3432 cm^−1^ and 3484 cm^−1^, the stretching vibration peaks of − CH_3_, −CH_2−_ were at 3000–2800 cm^−1^, and the wave number at 1160 cm^−1^ was the characteristic peak of C–O–C stretching vibration. The wave numbers at 952 cm^−1^ and 888 cm^−1^ corresponded to the stretching vibration peaks of C-O in the epoxy group. It could be seen that the addition of CSC has no effect on the structure of the cured product.

### DSC analysis

**Figure 3:**
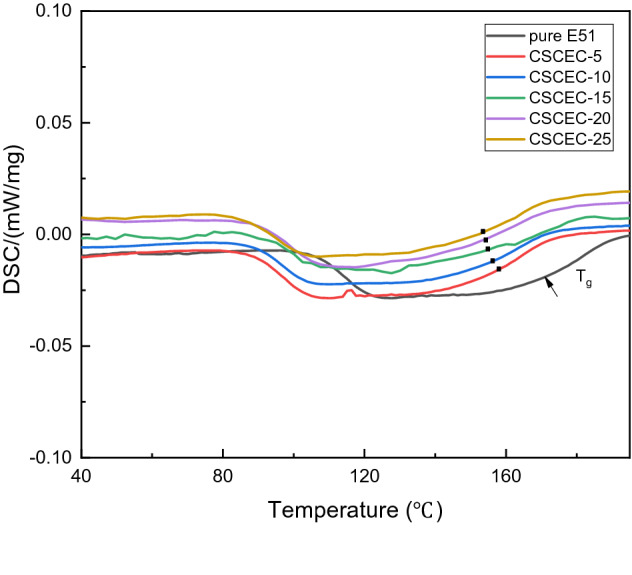
DSC curves of the cured pure E51 and the composites.

Figure 3 was the DSC curves of cured pure E51 and the composites. It could be seen from Fig. 3 that the T_g_ of pure E51 was 175℃ or so, which was higher than those of the composites. The possible reason was that the molecular dimension of CSC was smaller than that of E51, so that the end of chain of CSC was relatively more than that of E51. We have all known that the end of chain had more free volume. Once the CSC was added in the E51, the CSC provided more free volume for the E51 molecules which would make the chain segments of E51 move more easily.

### TG analysis

**Figure 4:**
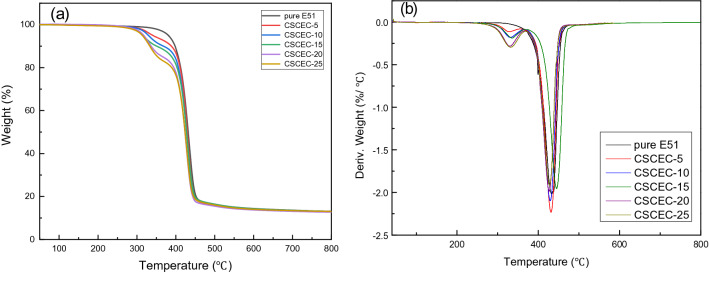
TG and DTG curves of pure E51 and the composites: (**a**) TG and (**b**) DTG.

From Fig. 4, for pure E51, until about 300 ℃, the chain segment motion of epoxy resin increased, some groups that were poor thermal stability began to decompose. With the increase of temperature, the forces between segments could not maintain the stability of the entire chain segment, the fracture occurred between segments. When the temperature reached 400 °C or so, the basic structure inside of the material was completely destroyed, a large number of thermal degradation occurred. At 500 °C, the thermal decomposition was almost complete.

The trend of thermal stability of CSC modified epoxy resin was the same as that of pure epoxy resin. It could be obviously observed in Fig. 4 that higher the CSC content was, lower the maximum thermal degradation temperature of the composites was. The main reason was that the thermal decomposition of CSC occurred at 250 °C, and the maximum thermal degradation temperature was around 350 °C. Therefore, in the temperature range of 300–400 °C, the thermal weight loss of the composites was more obvious than that of pure epoxy resin. After 400 °C, the thermal decomposition of CSC basically ended and CSC could no longer influence the thermal weight loss of the composites. Therefore, after 400 °C, the thermal weight loss of the composites was basically the same as that of pure epoxy resin. After 500 °C, thermal decomposition ended, the thermal weight loss did not change significantly and tended to be stable.

It could be seen from Table 1 that the T_5_ and T_10_ of the composites decreased significantly, which main reason was the low thermal decomposition temperature of CSC. Thereby, with the increase of CSC content, the decrease of T_5_ and T_10_ also increased.

**Table 1:** Thermogravimetric analysis data of pure E51 and the composites.

	T_5_/°C	T_10_/°C	DTG peak/°C	Y_c_ (%)
Pure E-51	380.76	398.14	434.03	13.1
CSCEC-5	340.85	388.51	430.51	12.7
CSCEC-10	329.55	368.07	430.04	12.9
CSCEC-15	321.67	347.42	429.72	13.1
CSCEC-20	317.59	336.53	428.89	12.7
CSCEC-25	313.91	333.07	426.52	13.0

T_5_ is the temperature when the thermal weight loss is 5%; T_10_ is the temperature when the thermal weight loss is 10%; Y_c_ is the char yield.

### Dynamic mechanical analysis

**Figure 5:**
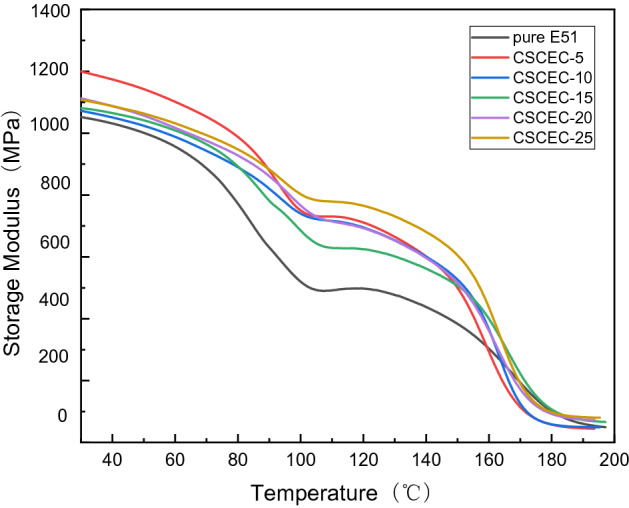
Storage modulus-Temperature curves of the composites.

As shown in Fig. 5, the storage modulus of each system decreased with the increase of temperature, and then it decreased sharply between 140 and 180 °C, which was the glass transition zone. The storage moduli of composites were higher than that of pure epoxy resin, especially before the glass transition. The initial storage modulus of pure epoxy resin is 1030 MPa, while the content was 5 wt%, the storage modulus of the composite reached 1174 MPa, which was increased by 14% compared with that of pure epoxy resin. This indicated that after adding CSC, the composites stored more energy in the elastic deformation process under the action of alternating stress. When the CSC content increased, the initial storage moduli were all lower than that of the content was 5 wt%, which might be because excessive CSC would cause agglomeration in the epoxy matrix, thus forming defects in the system.

**Figure 6:**
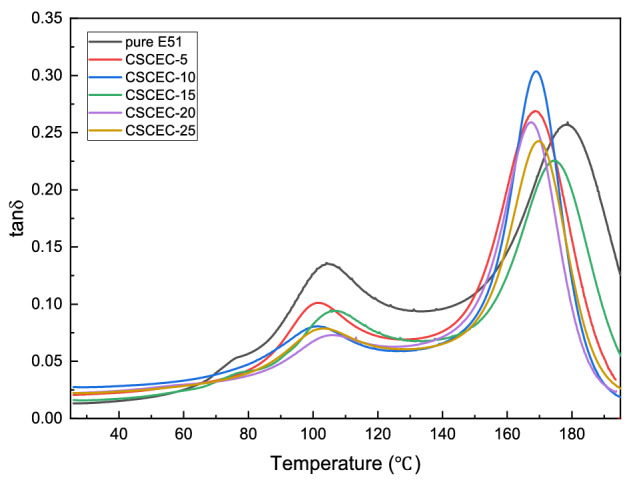
Tanδ-Temperature curves of the composites.

As could be seen from Fig. 6, the T_g_ of the composites was lower than that of pure epoxy resin. The T_g_ of pure epoxy resin was 178.7 °C, while the T_g_ of the composite was the lowest, 167.4 °C, when the CSC content was 20 wt%. The possible reason might be that the system was difficult to stir evenly when the amount of CSC exceeded 20 wt%, which resulted in this Tg’s trend. This trend was similar to that obtained by DSC.

It could be seen from Fig. 6 that there were two internal friction peaks in all curves, indicating that the curing crosslinking of the epoxy resin system was not uniform. As a result, there were local areas with different crosslinking densities inside the system. The tanδ peaks at low temperature were due to the presence of local structures with relatively low crosslinking density or even oligomers that had not been effectively cured, while the tanδ peaks at high temperature corresponded to local structural regions with high crosslinking density and difficult chain segment movement.

### Mechanical properties

#### Impact property

**Figure 7:**
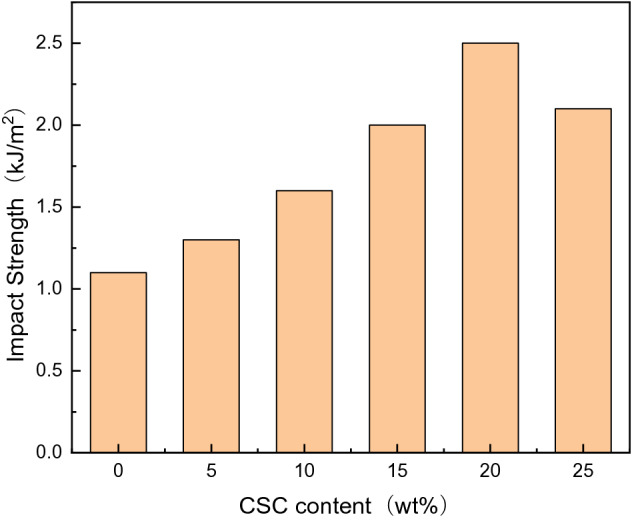
Impact strength drawing of the composites.

As could be seen from Fig. 7, the impact strengths of the composites were significantly improved compared with that of the pure epoxy resin, and increased first and then decreased with the increase of CSC content. The impact strength of pure epoxy resin was 1.10 kJ/m^2^, while the impact strength was the best when the CSC content was 20 wt%, reaching 2.50 kJ/m^2^, which was 127.2% higher than that of pure epoxy resin. As the CSC was added in the epoxy resin, CSC would be evenly dispersed in the matrix. Once the epoxy resin was subjected to external forces, the CSC might block the conduction of these external forces and absorb part of the external forces, thus improving the impact performance of the material. However, when the CSC content was too high, CSC might agglomerate in epoxy matrix, which resulted in the decrease of impact strength.

#### Tensile property

**Figure 8:**
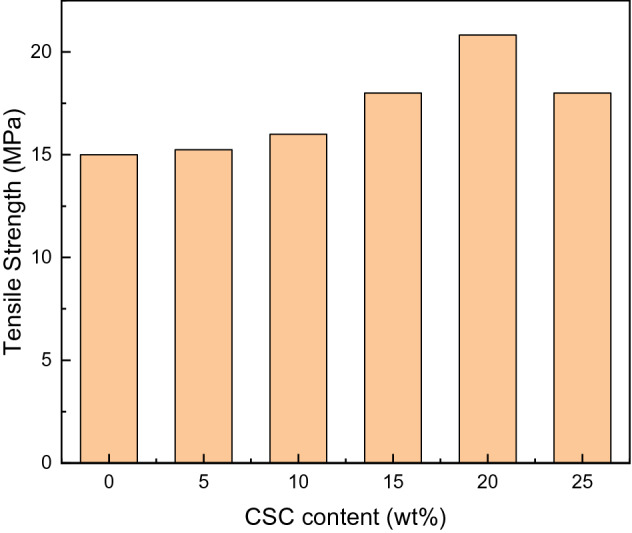
Tensile strength drawing of the composites.

Seen from Fig. 8, the variation trend of tensile strength with the increase of CSC content was similar to that of impact strength, which increased first and then decreased. The tensile strength of pure epoxy resin was 15.14 MPa, while the maximum tensile strength of the composites was 20.82 MPa when the CSC content was 20 wt%, which was 37.5% higher than that of pure epoxy resin. The reason was that only if the content was appropriate, the curing system might mix so well that more entanglements between the CSC and pure E51 were formed. The composites could take advantage of the strength of CSC to bear the stress, so that the CSC might deform in the process of stress transfer, which would disperse the stress and prevent the diffusion of cracks, thereby strengthening the composites. Once the CSC content was too high, CSC might agglomerate in epoxy matrix, which resulted in the decrease of tensile strength.

**Figure 9:**
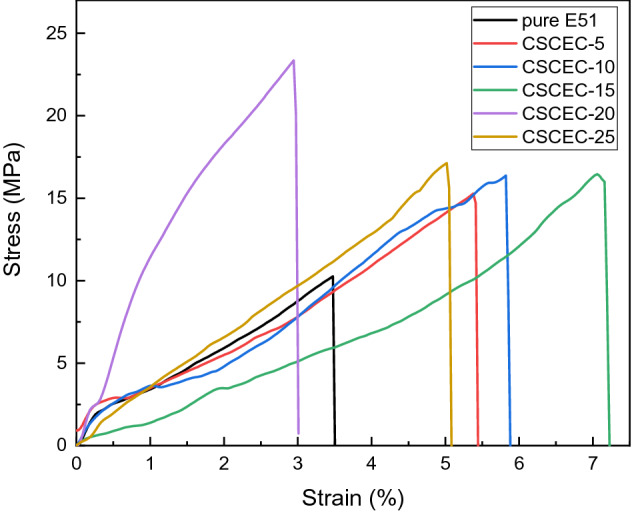
Tensile Stress–Strain curves of the composites.

Tensile stress–strain curves of the composites were seen in Fig. 9. When the external force acted, the force borne by the EP could be transferred to the CSC, which improved the tensile properties of CSCEC. According to the area of stress–strain curve in Fig. 9, the addition of CSC would improve the toughness of the composites in different degree.

#### Flexural property

**Figure 10:**
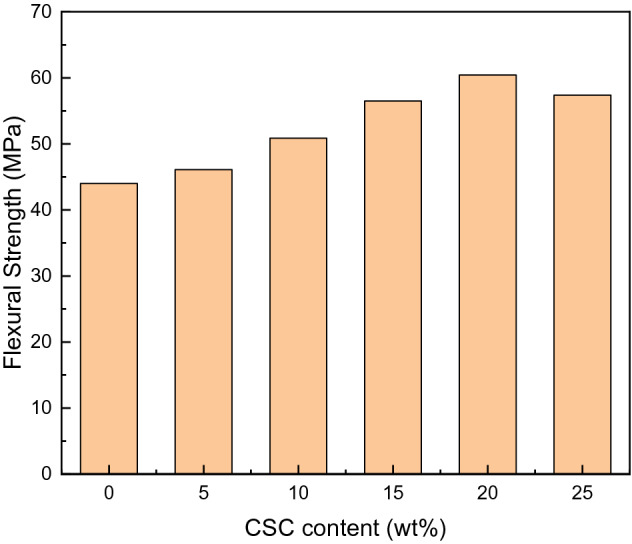
Flexural strength drawing of the composites.

It could be seen from Fig. 10, the trend of flexural strength with the increase of the CSC content was also similar to that of impact strength, increasing first and then decreasing. When the CSC content was 20 wt%, the maximum tensile strength of the composites was 60.43 MPa, which increased by 36.9% compared with pure E51. The reason was the same as tensile property that only if the content was appropriate, the curing system might mix so well that more entanglements between the CSC and pure E51 were formed. The composites could take advantage of the strength of CSC to bear the stress, so that the CSC might deform in the process of stress transfer, which would disperse the stress and prevent the diffusion of cracks, thereby strengthening the composites. Once the CSC content was too high, CSC might agglomerate in epoxy matrix, which resulted in the decrease of flexural strength.

### SEM analysis

**Figure 11:**
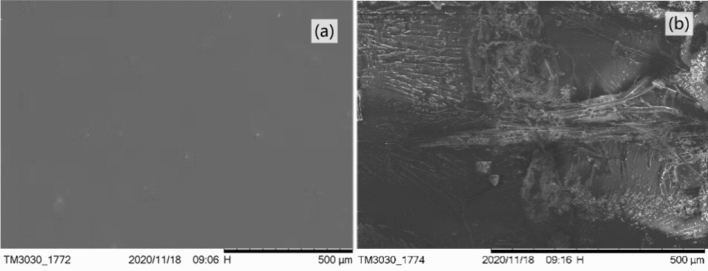
SEM image of impact fracture surface: (**a**) of pure E51; (**b**) of CSCEC-20.

The interface between CSC and epoxy resin could be studied by the impact fracture surface morphology of the composites. Figure 11a showed the morphology picture of the impact fracture surface of pure epoxy resin. The impact fracture surface was smooth which was a typical for the fracture of brittle material. Figure 11b showed the impact fracture surface morphology picture of the composite when the CSC content was 20%, which mechanical properties were the best. Compared with the pure epoxy resin, the fracture surface of the composite became obviously rough and had of obvious folds, which was a ductile fracture. As could be seen from Fig. 11b, a phase interface was formed between CSC and epoxy resin matrix, which main functions were to transfer stress, change the direction of the applied force on the interface to disperse it, stop and inhibit the growth of the cracks and absorb the impact energy. It showed that CSC could dissipate effectively the external stress on the composites and improve the toughness of the composites, which was consistent with the results of mechanical properties.

## Conclusions

When 2-MI was used as curing agent, CSC could effectively strengthen and toughen epoxy resin. When the CSC content was 20 wt%, compared with pure epoxy resin, the impact strength increased by 127.2%, tensile strength and flexural strength increased by 37.5% and 36.9% respectively. CSC could increase the energy storage moduli of the composites, and decrease the T_g_s of the composites. When the CSC content was 20 wt%, T_g_ is the lowest at 167.4 ℃ according to the DMA result. This trend was similar to that obtained by DSC. Due to the large amount of CSC, CSC would change the thermal degradation behavior of the composites markedly, and the T_5_ and T_10_ of the composites decreased significantly. The greater the amount of CSC, the more significant the decrease is. From SEM analysis, the fracture surface of the composite became obviously rough and had of obvious folds, indicating that CSC could toughen E51 effectively. In summary, CSC can strengthen and toughen E51, compared with other fillers that only strengthen or toughen epoxy resins.

## Data Availability

The datasets generated during and/or analysed during the current study are available from the corresponding author on reasonable request.

## References

[1] Zhang TT (2022). Multifunctional tannin extract-based epoxy derived from waste bark as a highly toughening and strengthening agent for epoxy resin. Indus. Crops Prod..

[2] Ma H, Aravand MA, Falzon BG (2022). Influence on fracture toughness arising from controlled morphology of multiphase toughened epoxy resins in the presence of fibre reinforcement. Compos. Sci.Technol..

[3] Karthikeyan L, Robert TM, Desakumaran D (2022). Epoxy terminated, urethane-bridged poly (ether ether ketone) as a reactive toughening agent for epoxy resins. Int. J. Adhes. Adhes..

[4] Xiao YL (2021). Construction of multifunctional linear polyphosphazene and molybdenum diselenide hybrids for efficient fire retardant and toughening epoxy resins. Chem. Eng. J..

[5] Zhang DJ (2013). Rheological properties of Al2O3 nanoparticle toughened epoxy resins. Adv. Compos. Lett..

[6] Zhao Q, Hoa SV (2007). Toughening mechanism of epoxy resins with micro/nano paticles. J. Compos. Mater..

[7] Ma HM (2016). Synthesis and research of epoxy resin toughening agent. Springerplus.

[8] Dai QZ, Chen JM, Huang Y (1997). Toughening of epoxy resin blended with rhermotropic hydroxyethyl cellulose acetate. J. Appl. Polym. Sci..

[9] Jung HS (2021). Caroline-Sunyong Lee, Evaluation of the mechanical properties of polyether sulfone- toughened epoxy resin for carbon fiber composites. Fibers Polym..

[10] Lee MY (2019). Fracture toughness of the novel in-situ polytriazolesulfone modified epoxy resin for carbon fiber/epoxy composites. J. Ind. Eng. Chem..

[11] Kwak NH, Lee JS, Ko NY (2019). Transparent epoxy resin toughened with in-situ azide-alkyne polymerized aliphatic toughening agent. Polym.-Korea.

[12] Lee B, Zhu J, Zhang RY (2018). Epoxy resins toughened with in situ azide-alkyne polymerized polysulfones. J. Appl. Polym. Sci..

[13] Liu WS (2014). From waste to functional additive: Toughening epoxy resin with lignin. ACS Appl. Mater. Interfaces.

[14] Kubouchi M, Hojo H (1993). Thermal-shock resistance of toughened epoxy-resins. Rubber.

[15] Li SY, Han XZ, Zhang QY (1997). Morphologies and mechanical properties of epoxy resins toughened by hydroxyl-terminated butadiene-acrylonitrile copolymer. Chem. J. Chin. Univ.-Chin..

[16] Xu J (2019). Bio-based hyperbranched toughener from tannic acid and its enhanced solvent-free epoxy resin with high performance. J. Renew. Mater..

[17] Suthan R, Jayakumar V, Gokuldass R (2021). Role of silicon coupling grafted natural fillers on visco0elastic, tensile-fatigue and water absorption behavior of epoxy resin composite. SILICON.

[18] Khan FM (2022). A comprehensive review on epoxy biocomposites based on natural fibers and bio-fillers: Challenges, recent developments and applications. Adv. Fiber Mater..

[19] Ridzuan MJM (2020). Effect of natural filler loading, multi-walled carbon nanotubes (MWCNTs), and moisture absorption on the dielectric constant of natural filled epoxy composites. Mater. Sci. Eng. B Adv. Funct. Solid-State Mater..

[20] Hrabe P (2022). Service life of adhesive bonds under cyclic loading with a filler based on natural waste from coconut oil production. Polymers.

[21] Miturska I (2020). The influence of modification with natural fillers on the mechanical properties of epoxy adhesive compositions after storage time. Materials.

[22] Zhang YJ (2021). Preparation of carboxylated lignin-based epoxy resin with excellent mechanical properties. Eur. Polym. J..

[23] Raju P (2020). Mechanical, wear, and drop load impact behavior of glass/Caryotaurens hybridized fiber-reinforced nanoclay/SiC toughened epoxy multihybrid composite. Polym. Compos..

[24] Chen B (2020). A novel and green method to synthesize a epoxidized biomass eucommia gum as the nanofiller in the epoxy composite coating with excellent anticorrosive performance. Chem. Eng. J..

[25] Dittenber DB, GangaRao HVS (2012). Critical review of recent publications on use of natural composites in infrastructure. Compos. A Appl. Sci.Manuf..

[26] Lou C, Zhou Y, Yan A, Liu Y (2022). Extraction cellulose from corn-stalk taking advantage of pretreatment technology with immobilized enzyme. RCS Adv..

